# Supplementary material for: Identification of Leishmania infantum Epidemiology, Drug Resistance and Pathogenicity Biomarkers with Nanopore Sequencing
Source: Microorganisms. 2022 Nov 14;10(11):2256. doi: 10.3390/microorganisms10112256 (PMC9697816; doi:10.3390/microorganisms10112256)
Supplement: Supplementary file 1 [file microorganisms-10-02256-s001.zip › microorganisms-1944861-supplementary.pdf]

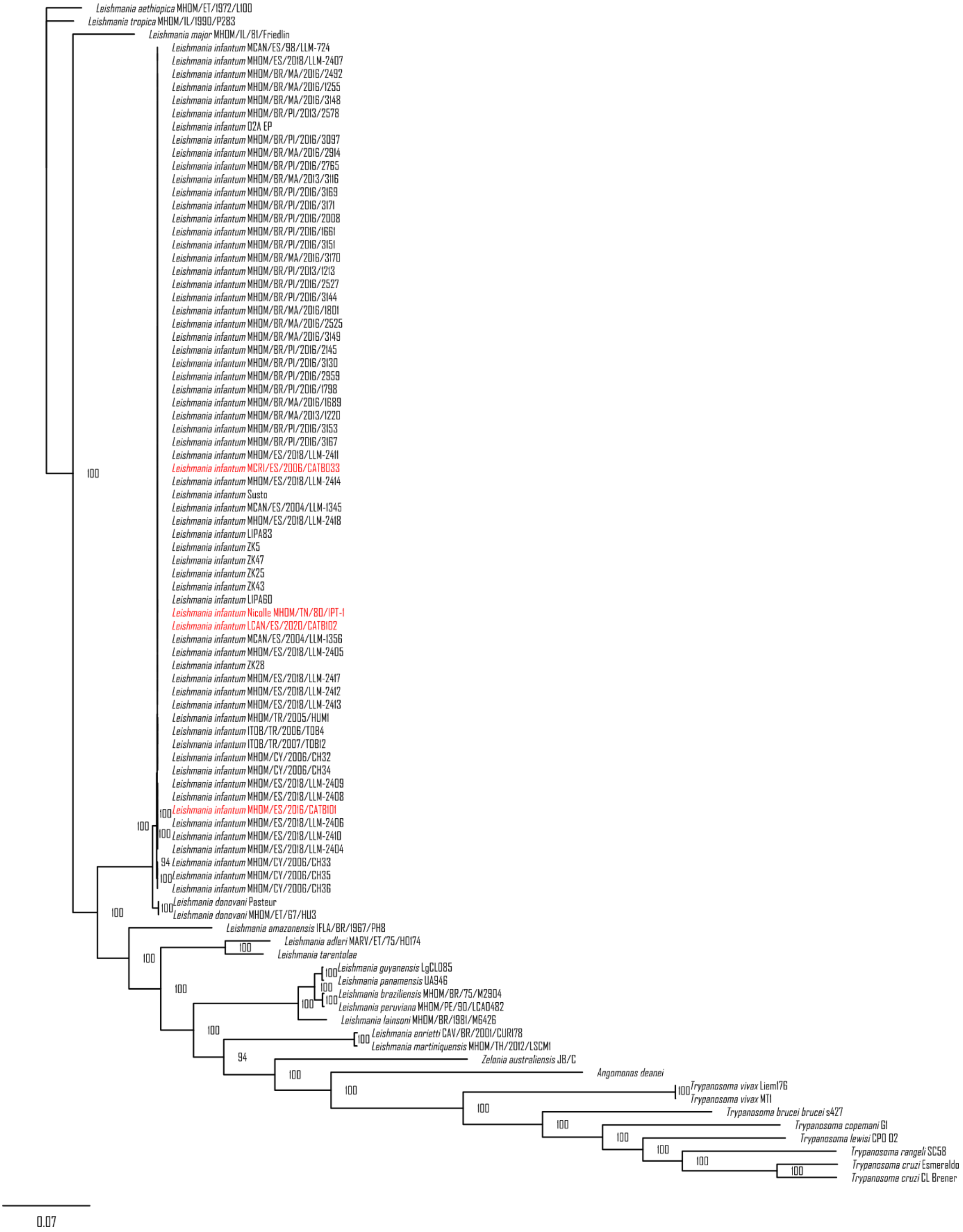

Supplementary Figure S1. Maximum likelihood phylogenetic tree of the *Leishmania* - *Trypanosoma* group. *Leishmania* - *Trypanosoma* consensus phylogeny was constructed using the conserved region of the maxicircle, concatenating the coding sequences of 12S rRNA, 9S rRNA, ND8, ND9, MURF5, ND7, CO3, CYb, ATPase 6, ND2, G3, ND1, CO2, MURF2, CO1, G4, ND4, G5 (ND3), RPS12 and ND5. The maximum likelihood tree (log-likelihood -163107.978) was modeled with the Tamura-Nei 1993 with 1,000 bootstraps. Taxa highlighted in red correspond to the novel maxicircle sequences of MHOM/TN/80/IPT-1, MHOM/ES/2016/CATB101, MCRI/ES/2006/CATB033 and LCAN/ES/2020/CATB102 isolates.
